# Epidemiology of COVID-19: What changed in one year?

**DOI:** 10.3906/sag-2110-153

**Published:** 2021-12-17

**Authors:** Cemal BULUT, Yasuyuki KATO

**Affiliations:** 1 Department of Infectious Diseases & Clinical Microbiology, Faculty of Medicine, Health Science University, Ankara Turkey; 2 School of Medicine, International University of Health and Welfare, Narita Japan

**Keywords:** COVID-19, epidemiology, pandemic

## Abstract

A coronavirus brought the first pandemic attack of this century as a flu virus did a hundred years ago. This greatest pandemic of the century brings us new opportunities to understand and explore the dynamics of a contagious disease. Nearly two years later, we are still collecting the evidence to understand the disease. Some basic epidemiological properties are still urgently needed. Not only the origin of the virus but also Ro value, possible transmission routes, epidemiologic curves, case fatality rates, seasonality, severity and mortality risk factor, effects on the risk groups, differences between countries and so on still require strong evidence prior to making final suggestions. In this review, we tried to evaluate the epidemiological evidence to scrutinize where exactly we are in this pandemic.

## 1. Introduction

A coronavirus brought the first pandemic attack of this century as a flu virus did a hundred years ago. Nowadays, this greatest pandemic of the century greatly opens up new opportunities for our lives to understand and explore the dynamics of a contagious disease in detail. Almost two years later, we are still collecting the evidence to clearly comprehend the disease. With this respect, some basic epidemiological properties are still questioned due to several unknown parameters in this area. In these cases, scientific findings should be supported and augmented theoretically and experimentally during/after the pandemic. In this study, we aim to evaluate epidemiological evidence to obviously elucidate where exactly we are in this pandemic.

## 2. Origin of the virus

It is well-known that bats are reservoirs of coronaviruses. It is interesting to note here that most of these viruses are unknown to us and their number might be 1200–6000 [1]. A study focusing on the evolutionary origin of SARS-CoV reported four novel SARS-CoV-2–related viruses. Another result of this report was that high diversity of bat coronaviruses could be placed in a small area [2]. 

Even though reported new bat viruses highly related to SARS-CoV or SARS-CoV-2 in China and neighboring countries strengthen the theory of zoonotic origin [3], similarity between SARS-CoV-2 and viruses studied at the Wuhan Institute of Virology does not remove the questions about a laboratory leak incident, as reported elsewhere [4,5].

According to the WHO report about the origin of SARS-CoV-2 virus, for introduction of virus, possible-to-likely pathway is direct zoonotic spillover. Keep in mind that the existence of an intermediate animal host for this introduction had been considered likely to very likely pathway. Furthermore, possibility of a laboratory escape from a laboratory working with animal coronavirus had been reckoned as extremely unlikely pathway [6]. 

## 3. Ro and Rt value of COVID-19

Basic reproduction number (Ro) as an indicator of the transmissibility of a virus is utilized frequently. Ro of COVID-19 was quite different among countries. It is well-known that its value was 3.2 in China whilst it was 2.2 in western European countries [7]. In a mathematic modeling, Ro values in Turkey and Japan were 1.71 and 4.3, respectively [8]. In a metaanalysis, the pooled global Ro was found to be 4.08 [9]. Stated simply, the estimated summary reproductive number was 2.87 (95% CI, 2.39–3.44) [10]. As for time-varying reproduction number (Rt), it is the value for the population which was not completely susceptible and not fully adopted some prevention or control measures. In a study, upon evaluating Rt values for 160 countries, it can be expressed that this value was nearly 10 at the beginning of pandemic [11]. 

## 4. Incubation period

Incubation period of COVID-19 was 5.7 days according to a systematic review result [12]. This period could be as short as 3.1 days and could be long as 12.1 days. It can be seen that incubation period was a bit shorter than one year before report [13,14]. The surprising result in [20] was that the incubation period in men could be about 3.2 days longer than in women. This period seems to be different in several countries [12].

## 5. Epidemic curves of COVID-19

**Figure 1:**
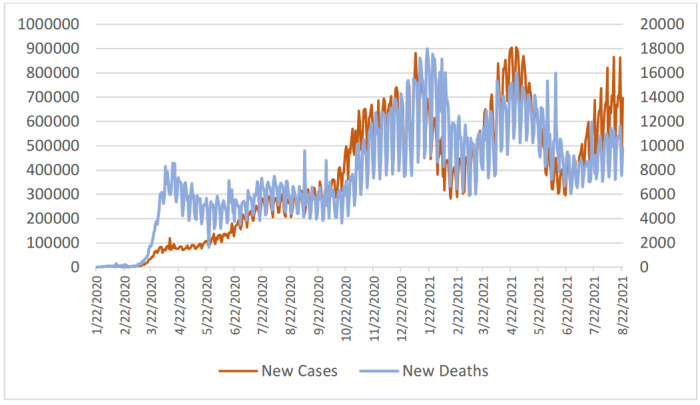
New daily case and death numbers all over the world.

**Figure 2:**
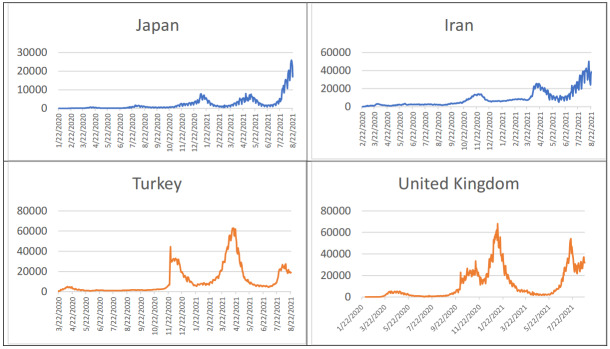
New daily case numbers of some countries from different parts of the world.

As of June 21, according to WHO data, COVID 19 has been confirmed as the cause of a total of 180,518,201 cases and 3,918,120 deaths. Many areas of the world have experienced four series of waves of epidemic (see Figure 1 for details). For some countries such as Iran and Japan, each wave was bigger than previous ones, while for others such as Turkey and United Kingdom the amplitudes of waves were getting low, as seen in Figure 2.

**Figure 3:**
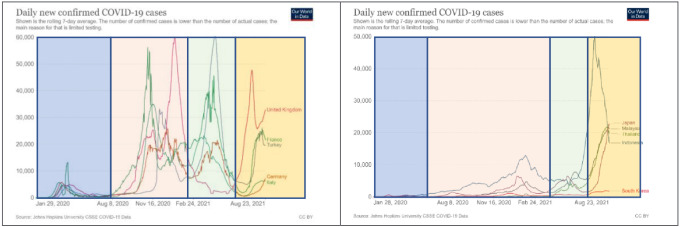
Epidemic curves of some European and Asian country and waves of outbreak.

These epidemic curves have been partly formed by strong restriction rules and preparedness of the health care systems. Surprisingly, its shapes were also quite different in the Western and the Eastern parts of the world. It is obviously seen in Figure 3 that whilst in Europe, big and devastating waves occurred during the first year of the pandemic, eastern Asian countries saw the highest numbers of case and death during the second year of the disease. 

Different scenarios such as progression rate, mortality, and Ro values were noted for each country, and each country developed their own new normal rules to overcome the pandemic. It seems that progression of pandemic was affected not only by governments’ political and economic decisions or accessibility to health care, but also by seasonality, weather condition, vaccination status, and many different specified or unspecified factors. 

## 6. Transmission routes

### 6.1. Respiratory routes

As other respiratory pathogens, SARS-CoV-2 spreads mainly by respiratory secretion produced by cough, sneeze, and talk. In spite of the fact that person-to-person contact is the major route of transmission, various sizes of contaminated air particles seem to be involved [15]. It is widely accepted that SARS-CoV-2 virus is primarily spread by large droplets (>5 µL). Nevertheless, more scientific evidence has been available supporting that smaller particles transmit the virus and airborne transmission can occur [16]. Although live viruses were isolated from surface and air samples, their half-live was around 6 h [17]. Additionally, Ro value of SARS-CoV-2 is very low as compared to other airborne transmitted viruses. More importantly, another exact fact is that aerosol generating procedures could lead to aerosol transmission and could be problematic in hospital settings [17]. 

### 6.2. Direct contact 

The presence of SARS-CoV-2 in the environment and contamination of the surface have been recognized as a possible mode of transmission of COVID-19. This environmental contamination could be a problem especially in health care settings [18]. It seems that durability of SARS-CoV-2 on different materials is a bit shorter than other coronaviruses [19]. Environmental contamination could be as high as 54%–69% of the contact surfaces, with SARS-CoV-2 loads ranging from 28.1 to 132.7 gene copies per cm^2^ [20]. Though environmental contamination serves as a possible route transmission, the contribution of this type of transmission remains unclear [18].

### 6.3. Vertical routes (mother to children)

It is well-known that maternal SARS-CoV-2 infection could increase the risk of some respiratory disorders and could cause some other morbidities in neonates [21]. However, vertical transmission had not been demonstrated as a transmission route [22]. Anecdotal detection of SARS-CoV-2 in the fetal site of placenta by using different methods had brought a possibility of transplacental transmission and fetal infection [23]. The probability of vertical transmission is not as high enough as it was at the beginning of the pandemic [24-26]. 

### 6.4. Oral–fecal routes

Although the detection rate of SARS-CoV-2 RNA in fecal samples is high, the isolation rate of viable viruses seems low. In a study from China, serial stool sampling showed that viral shedding was directly related to disease severity and it could be as long as 5 weeks [27]. This study also reported a successful isolation of SARS-CoV-2 in 2 samples collected (when?). Another important point was more than 2/3 of the patients had positive fecal RNA after pharyngeal swabs became negative [28]. Some other reports suggested that environmental sampling of sewage water revealed the presence of SARS-CoV-2 and relevant community outbreaks of COVID-19 [29]. These reports raises a new possibility that oral–fecal transmission of SARS-CoV-2 could occur like the outbreak of SARS in Amoi Garden occurred through the sewage system in 2003 [30].

## 7. Asymptomatic carriage/infection 

According to a metaanalysis, up to half of COVID patients could be asymptomatic. The rate of this condition could be higher among female and children [31]. It is interesting to note that an earlier metaanalysis showed that asymptomatic infection rate was as low as 16% [32]. Results of another metaanalysis indicated that these asymptomatic cases could transmit the virus for a longer period and could have subclinical lung injuries [33]. Nonetheless, some other studies emphasized the need for a standard asymptomatic infection definition [34]. It is heartening to note that these metaanalyses also showed the need of early recognition, better surveillance, and effective preventive strategies to control COVID-19 disease in population [32-36].

## 8. Superspreaders

The possibility of infecting a large number of patients has become an attractive topic of COVID-19. Patients who infect a large number of susceptible people are accepted as superspreaders. Characteristics of superspreaders remain unclear. Predisposing conditions like environmental conditions, large gatherings, ineffective ventilation systems, unwise use of personnel protective equipment, poor hygiene are among possibilities, but these questions have not been properly answered yet [37]. Recent reports from different countries have showed that 10%–20% of the cases were responsible for 80% of transmission [38], which suggests that superspreading events occur more commonly than previously thought. 

## 9. Reinfection

Emergence of antigenic variation is a challenge for control of respiratory viruses [39]. Coronaviruses has become an example of these challenges. In spite of the fact that SARS-CoV-2 generates enough B cell and T cell response to control the disease, lack of long-term immunity is an accepted reality for coronavirus-related immunity [40]. New evidence exhibits that immunity could not wane for a year at least [41]. A review showed that reinfection rate was very low (0.0%–1.1%) and there was no evidence of increasing risk of infection over time [42]. Another clear fact was that reinfection rate was higher in younger patients, and generally, disease severity was not increased in the second course [43,44]. However, we need more detail about if these results are due to viral reactivation, reinfection, or false test results [43].

## 10. Seasonality of COVID-19

In general, all respiratory viruses have seasonal cycles [45]. These cycles are formed both by environmental factors and human behavior. Newly discovered SARS-CoV and SARS-CoV-2 infections also started in winter months. In addition, some reports support the seasonal nature of SARS-CoV-2 [46,47]. However, it cannot be pointed out that climatic parameters such as temperature or humidity alone play a central role during the pandemic [48]. Therefore, it is important to point that countries should focus on health policies rather than weather variable to control the pandemic.

## 11. Mortality, how deadly COVID-19 is?

**Figure 4:**
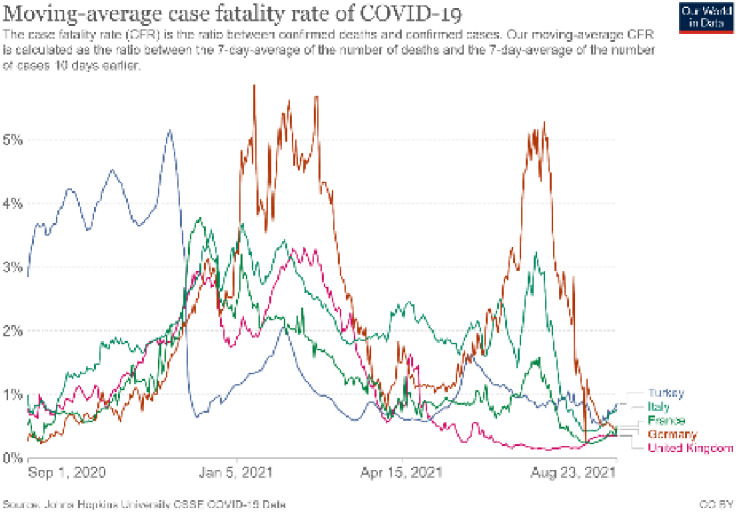
Epidemic curves of some European and Asian country and waves of outbreak.

The overall case fatality of COVID-19 was 2.1% at the end of August 2021 World Health Organization (2021). COVID-19 Weekly Epidemiological Update, Edition 55, published 31 August 2021 https://www.who.int/publications/m/item/weekly-epidemiological-update-on-covid-19---31-august-2021 [Access data:01.09.2021]. Nevertheless, this rate was 6.3 on the April 2020 World Health Organization (2020). Coronavirus disease 2019 (COVID-19). Situation Report – 84. https://www.who.int/docs/default-9 source/coronaviruse/situation-reports/20200413-sitrep-84-covid-19.pdf?sfvrsn=44f511ab_2 [Access data:14.07.2021] . Herein, it seems that the mortality rates have shown great differences among patient groups and between countries. Case fatality rates in several countries are presented in Figure 4, which shows different case fatality rates observed at different phases of the pandemic.

It is noticed that mortality rates of COVID-19 vary widely in different countries. Generally speaking, at the end of August 2021, this number fell in the range of 0.46 and 9.24. Crude mortality rates of some countries whose case number is greater than a hundred thousand are listed in Table 1.

**Table 1:** Crude mortality rates of some countries (August 2021).

Name	Cases, cumulative total	Deaths, cumulative total	Crude mortality rates %
Peru	2,142,153	197,879	9.24
Mexico	3,225,073	253,155	7.85
Ecuador	498,678	32,087	6.43
China	122,744	5676	4.62
Tunisia	642,788	22,609	3.52
Zimbabwe	123,001	4293	3.49
Indonesia	4,008,166	128,252	3.2
South Africa	2,698,605	79,584	2.95
Italy	4,488,779	128,795	2.87
Brazil	20,570,891	574,527	2.79
Namibia	123,861	3345	2.7
Russian Federation	6,785,374	177,614	2.62
Viet Nam	358,456	8666	2.42
Germany	3,877,612	92,022	2.37
Chile	1,634,394	36,688	2.24
Pakistan	1,127,584	25,003	2.22
Iran	4,715,771	102,648	2.18
Argentina	5,133,831	110,352	2.15
The United Kingdom	6,524,585	131,680	2.02
Kenya	229,628	4528	1.97
France	6,448,367	112,180	1.74
Spain	4,794,352	83,337	1.74
United States of America	37,588,957	623,900	1.66
India	32,474,773	435,110	1.34
Japan	1,318,346	15,663	1.19
Iraq	1,832,240	20,262	1.11
Republic of Korea	239,287	2228	0.93
Malaysia	1,572,765	14,342	0.91
Thailand	1,083,951	9788	0.9
Turkey	6,234,520	54,765	0.88
Cuba	592,619	4618	0.78
Israel	1,005,511	6864	0.68

Table 2 summarizes and compares the changes on mortality rates of selected countries during the pandemic. To explain in more detail, it can be stated that, as given in Table 2, an interesting result was that while some countries that had higher mortality rates in the first months of the pandemic, a decline in the rate was observed in later phases of the pandemic. Another interesting point was that some countries with higher mortality rates in the early months of the pandemic recorded a decline in the rate at the later stages of the pandemic (Table 2).

**Table 2:** Changes on mortality rates of selected countries during the pandemic.

Countries	June 30, 2020	December 31, 2020	March 31, 2021	August 15, 2021
Bangladesh	1.3	1.5	1.5	1.7
Brazil	4.3	2.6	2.5	2.8
Canada	8.3	2.7	2.3	1.8
France	18.9	2.5	2.0	1.7
Germany	4.6	1.8	2.7	2.4
India	3.0	1.4	1.3	1.3
Indonesia	5.1	3.0	2.7	3.1
Iran	4.7	4.6	3.3	2.2
Iraq	3.9	2.2	1.7	1.1
Italy	14.5	3.5	3.1	2.9
Japan	5.2	1.5	1.9	1.3
Mexico	12.3	8.9	9.1	8.0
Pakistan	2.1	2.1	2.2	2.2
Peru	3.3	3.7	9.3	9.3
Republic of Korea	2.2	1.4	3.4	3.0
South Africa	1.8	2.7	1.7	1.0
Spain	11.4	2.7	2.3	1.8
Turkey	2.6	1.5	1.0	0.9
United Kingdom	14.0	3.1	2.9	2.1
United States of America	5.0	1.8	1.8	1.7

## 12. Risk factors for mortality and severe diseases

Now, many risk factors for mortality and severe disease have been clearly defined. According to metaanalyses, some chronic comorbidities, demographic variables and laboratory findings are identified as risk factors for higher mortality. 

Chronic comorbidities, complications, and demographic variables including acute kidney injury, COPD, diabetes, hypertension, cerebrovascular disease, cancer, increased D-dimer, male sex, older age, current smoking, and obesity are clinical risk factors for a fatal outcome associated with COVID-19 [49-51]. Another finding of the metaanalyses was that there was considerable variety in the prevalence of comorbidities, and severe disease and mortality in different geographic regions. While the prevalence of comorbidities was highest in the US studies, the proportion of severe disease of COVID-19 was highest in Asian studies and the mortality was highest in the European and Latin American countries [52].

Potential genetic host factors like HLA-C*04:01, HLA-A*01:01, HLA-A*02:01, and HLA-A*03:01 have been identified as having an important role in immune defense against COVID-19 [53,54].

Prognostic score, a combination of variables like age, comorbidity, CD4+ T cell count, C-reactive protein (CRP), D-dimer, lactate dehydrogenase, cardiac troponin I, have been developed to predict the progression to severe illness and death in COVID-19 patients [55,56]. These scores should be applied carefully to different situations and countries.

## 13. Conclusion 

We have achieved better understanding of the epidemiology of COVID-19 in the past 2 years. However, we still do not know when this pandemic will finally end. We need to create more evidence and make further scientific progress to understand, to explore, and to pass on the future generations what exactly happened during this pandemic and how we acted to control it.
